# Utility of perfusion decellularization to achieve biochemical and mechanically accurate whole animal and organ‐specific tissue scaffolds

**DOI:** 10.14814/phy2.14804

**Published:** 2021-03-26

**Authors:** Daniel Shiwarski

**Affiliations:** ^1^ Department of Biomedical Engineering Carnegie Mellon University Pittsburgh PA USA

Due to the limited supply of donor organs and the need for new sources of replacement tissue, researchers continue to strive for new ways to engineer partial and whole organ replacements. A key step is to recreate the 3‐dimensional (3D) architecture using extracellular matrix (ECM)—with tissue‐specific composition—to provide the mechanical and biochemical cues required to promote tissue development. To address the challenge of creating 3D tissue scaffolds, the field has turned toward tissue decellularization as a source for tissue‐specific ECM and as a way of obtaining a cell‐free scaffold for tissue engineering and regenerative medicine.

The general idea is to remove the cellular material to avoid potential cross‐species immune response and adverse reactions during implantation. The goal of the decellularization process is to create a tissue‐specific ECM scaffold from donor tissue that can be used to promote wound regeneration and possibly begin to create de novo organs. To date, decellularized tissue has been widely used and shown to promote better regeneration for nerve conduits (Hudson et al., 2004; Sarker et al., 2018), improve cellular adhesion and bioactivity (Hussey et al., 2018; Wolf et al., 2012; Zhang et al., 2016), and can be used for recellularization as a way to introduce patient‐specific cells into an organ‐derived ECM scaffold (Ott et al., 2008). More recently, decellularized tissues and organs have been utilized as an ideal complex ECM biomaterial for advanced 3D tissue engineering (Jung et al., 2016; Pati et al., 2014).

Most decellularization processes begin by removing the tissue or organ of interest from a mouse, rat, or other animal, using standard detergents such a SDS and Triton X‐100 to slowly remove cells from the tissue via submersion or perfusion (Crapo et al., 2011). Submersion decellularization is limited to thin tissue samples and tends to be inadequate for whole organ decellularization (Crapo et al., 2011). Perfusion decellularization requires intact vasculature to penetrate the internal tissue structure and enables complete tissue decellularization and subsequent recellularization (Crapo et al., 2011). One potential downside of standard perfusion decellularization processes is the requirement for organ dissection and removal from the animal. In this recent study, Taylor et al. have demonstrated a novel whole animal perfusion decellularization approach that can be used to obtain a completely decellularized rat or can be used to isolate organ systems and specific tissues in a way that maintains both the mechanical integrity and tissue‐specific ECM composition of the organ (Taylor et al., 2021).

This manuscript uniquely leverages the advantages of perfusion decellularization to demonstrate whole body decellularization of a rat. This is an impressive technical feat which highlights the utility in using native vascular networks to perform decellularization and shows that with their approach any solid organ can be decellularized in situ. The downside to this whole animal approach, as mentioned by the authors, is the 7–9 days of perfusion required to achieve translucent organs (Taylor et al., 2021). Additionally, following whole animal decellularization, the organs or organ systems require subsequent removal to confirm decellularization and for use as future scaffolds or ECM biomaterials. This reduces the need for a whole animal decellularization approach, and in some cases could even add unnecessary processing steps and experimental complexity. To reduce decellularization time and achieve organ‐specific decellularization, Taylor et al. excised organs and organ systems using the same methods demonstrated for the whole animal (Taylor et al., 2021). Remarkable decellularization was achieved in both neonatal and adult rats for the abdominal and thoracic compartments, kidney, liver, heart lung pair, and even a lower limb. The vascular networks within these organs and organ systems were intact after decellularization and would likely serve as an ideal conduit for recellularization in future experiments (Taylor et al., 2021).

In addition to demonstrating broad applicability in their decellularization method for solid organs, Taylor et al. performed a structural, proteomic, and mechanical evaluation of the decellularized ECM (Taylor et al., 2021). This is important for confirmation that their approach does not eliminate the organ‐specific proteins, microstructure, and material properties from the tissue when removing the cells. It also highlights the structural complexity that is left behind such as the glomerulus seen in their scanning electron micrographs of a decellularized kidney. By providing a table of residual ECM proteins from their decellularized heart, kidney, and liver, they have both shown the diversity of the ECM proteins remaining in their scaffolds and created a useful resource for the field to reference when determining important ECM protein distinctions between various organs (Taylor et al., 2021). This resource is particularly useful for the field of ECM material development for tissue engineering and regenerative medicine where researchers are often attempting to recreate the most accurate 3D environment to promote cell differentiation, native tissue mechanical strength, and recreate organ physiological function.

This report is a useful addition to the growing field of tissue decellularization by highlighting the simplicity and breadth of perfusion decellularization to produce whole animal and isolated organ system scaffolds in a rat that maintains the tissue ECM protein complexity and mechanical properties. As the field of regenerative medicine and tissue engineering continues to move away from 2D toward 3D platforms as a way to recapitulate the mechanical and biochemical complexity of tissue, methods for efficient decellularization are essential. Scaffolds generated via this approach will likely serve as ideal biomimetic 3D environment for recellularization and can be homogenized and acidified to form the base biomaterial for advanced 3D biofabrication techniques such as 3D bioprinting.
